# Pull-Out Bond Strength of Titanium Post Cemented with Novel Fast-Setting Calcium Silicate Cement

**DOI:** 10.14744/eej.2021.69875

**Published:** 2021-11-23

**Authors:** Bahram RANJKESH, Mariantonietta LEO, Ali VAFAEI,, Henrik LØVSCHALL

**Affiliations:** 1. Section for Prosthetic Dentistry, Department of Dentistry and Oral Health, Aarhus University, Denmark; 2. Department of Clinical Sciences and translational Medicine, University of Rome Tor Vergata, Roma, Italy; 3. Department of Pediatric Dentistry, Faculty of Dentistry, Tabriz University of Medical Sciences, Tabriz, Iran; 4. Section for Oral Ecology and Caries Control, Department of Dentistry and Oral Health, Aarhus University, Denmark

**Keywords:** Calcium silicate cement, cementation, endodontically-treated teeth, prefabricated intracanal post, retentive strength

## Abstract

**Objective:**

The aim of this study was to compare the pull-out bond strength of prefabricated titanium posts cemented with novel fast-setting calcium silicate, zinc phosphate, or glass ionomer cements.

**Methods:**

Sixty extracted human maxillary incisors were selected and received root canal treatment. Post space was prepared for titanium ParaPost XP size 5 (diameter=1.25 mm). The posts were cemented using novel calcium silicate cement, zinc phosphate cement, or glass ionomer cement (n=20). Specimens were stored in phosphate-buffered saline for 4 weeks. Subsequently, the posts were subjected to axial tensile force until bond failure. Data were analyzed by one-way ANOVA followed by multiple comparisons.

**Results:**

The posts cemented with novel calcium silicate cement (10.5±3.8 MPa) demonstrated significantly higher bond strength than zinc phosphate (8.0±2.6 MPa) and glass ionomer cements (8.0±2.7 MPa) (P<0.05).

**Conclusion:**

Within the limitation of the study, the pull-out bond strength of titanium post cemented with novel calcium silicate cement in endodontically treated teeth was superior to zinc phosphate and glass ionomer cements.

## Introduction

Restoration of severely damaged teeth due to caries or trauma may often require insertion of intracanal posts to achieve sufficient mechanical retention (1). The space preparation for insertion of the post inside the root canal results in an unavoidable removal of root dentine that may eventually weaken the remaining tooth structure (2). However, knowing that the risk of root fracture increases as a result of root canal treatment (3) and intracanal post placement (4), insertion of posts is often an unavoidable alternative. Intracanal post systems are available in various materials, forms, and surface textures. Prefabricated metal posts either passively cemented or actively screwed into the root canal have been used for years (5). Endodontically-treated teeth restored with prefabricated metal posts have shown favourable long-term prognosis in the clinical service over 10 years (6, 7). Although active posts are more retentive than passive posts, they propose more stress into the root dentine escalating the root fracture risk; thereby cemented serrated posts are most desirable (8). The cement type affects the retention of the posts since it plays as a bonding media between post and dentine (9). Furthermore, the cementing agent should not negatively influence the sealing of the root canal system (10), and the outcome of the root canal treatment.

**Highlights**

• Novel fast-setting calcium silicate cement with fluoride has been developed for potential applications in tooth crown beside the traditional application of calcium silicates in root.
• The fluid consistency of the novel cement, by slight adjustment of powder-to-liquid ratio, allows the application of the cement as the luting agent for intracanal posts.
• The bond strength of cemented titanium intracanal posts with novel calcium silicate cement was significantly higher than zinc phosphate and glass ionomer cements.

Interestingly, calcium silicate cements, which have been used for bioactive sealing of root dentine, have also been further innovated to improve mechanical properties and handling properties. Mineral trioxide aggregate (MTA) as the first generation of calcium silicate cements in dentistry is well known to provide an excellent sealing (11), with outstanding biocompatibility (11), and apatite-forming ability in physiological-like environments (12, 13) that enhances the long-term bonding to dentine (14). However, lower initial mechanical strength (15), long setting-time (16), and particularly poor handling characteristics with sand-like consistency (17) limit MTA application as cementing agent. Only one study has reported the superiority of zinc phosphate or glass ionomer compared to MTA when used as luting agents for posts in root canal treated teeth (18).

Novel fast-setting calcium silicate cement (Protooth) with a fluoride additive and zirconium oxide as the radiocontrast element has been developed for potential applications in tooth crowns (12, 19-21). The consistency of the novel cement, from fluid to condensable, is controllable with adjusting the powder-to-liquid ratio. The initial setting time ranges from 4 to 6 minutes at thick consistency and 8 to 10 minutes at creamy consistency. The novel cement is white and comprises the same components as the original MTA (Table 1). The Protooth formulation has higher mechanical properties than other calcium silicate cements (ProRoot MTA and Biodentine) (19) with the ability to support superficial apatite deposition when immersed in phosphate-buffered solution (12). The apatite-forming ability of Protooth endorses closure of the experimental gaps at the cement-dentine interface (20), and improves its bonding to dentine over time (21). Biocompatibility of Protooth has been documented *in vitro* (22) and *in vivo* (23-25). A novel version of fast-setting calcium silicate cement here called Bond Protooth, which does not contain radiocontrast is a candidate material for application as cementation agent.

**Table 1.** The composition of the tested materials in the study

Cement (manufacturer)	Composition
Novel calcium silicate cement	Tricalcium silicate, dicalcium silicate, tricalcium aluminate, and calcium sulfate (calcium- (Protooth, Dentosolve, Aarhus, Denmark) silicate-aluminate composition: CaO 60-70%, SiO_2_ 20-30%, Al_2_O_3_ <5%, tricalcium aluminate >7%, SO_4_ <3%, additive fluoride (3.5% wt.), nano-silica, and PO_4_ (Patent Pub. No. WO 2011/023199)
Zinc phosphate cement (DeTrey, Dentsply, Germany	Base paste: (1,3-butylene glycol Disalicylate, zinc oxide, calcium phosphate, calcium tungstate, and iron oxide pigments) Catalyst paste: (calcium hydroxide, N-ethyl-o/p-toluene sulphonamide, zinc oxide, titanium oxide, zinc stearate, and iron oxide pigments)
Glass ionomer cement (Fuji I, GC, Tokyo, Japan)	Fluoro-aluminosilicate glass, polybasic carboxylic acid

The aim of this study was to evaluate the bond strength of prefabricated titanium post cemented with Bond Protooth compared to zinc phosphate and glass ionomer cement. The null hypothesis was there was no difference in bond strength of cemented metal post into root canal using the three cement types.

## Materials and Methods

Sixty extracted single-rooted human maxillary incisors, kept in 0.4% thymol, were selected and mechanically cleaned. The methodology was adopted from the study by Vargas et al. (18). Assuming the mean retentive value in glass ionomer group as 360 Newton (18) with 20% increase expectation in novel calcium silicate group with 20% deviation (obtained from the pilot study), and power of 80% at significance level of 5%, the sample size calculation revealed 20 samples in each group. The crowns were removed at cementoenamel junction using diamond disk (Struers, Ballerup, Denmark) under water coolant. Only circular-shape canals were included in the study. The remaining root was measured and roots were assorted to five root length subgroups ranged from 15 to 19 mm with 1 mm increments. Root canal treatment was performed after negotiating the patency and establishment of working length 1 mm shorter than root length measured by visual detection of #10 K-file through the major apical foramen. The root canals were instrumented using NiTi rotary file system from S1 to F2 (ProTaper file system, Dentsply Maillefer, Ballaigues, Switzerland) and irrigated with 5.25% NaOCl between the files. The smear layer was finally removed using 15% EDTA for one minute. The canals were obturated with single cone gutta-percha point (F2 gutta-percha, ProTaper, Dentsply Maillefer, Ballaigues, Switzerland) and root canal sealer (AH Plus, Dentsply Sirona, Ballaigues, Switzerland). The teeth were kept in a moist environment at 37°C. After 7 days, 10 mm gutta-percha was removed from the canal using Gates-Glidden bur #4. The post space was eventually prepared using ParaPost XP drill #5 (Coltene Whaledent, USA). The canals received following irrigation: 1 minute 17% EDTA, saline irrigation, 1 minute 5.25% NaOCl, and finally saline irrigation (18). Teeth were individually embedded in cold-cure poly(methyl methacrylate) (Major.Skel; Major Prodotti Dentari, Moncalieri, Italy) in a perpendicular position. Thereafter, the specimen in each root length subgroups were randomly divided into three groups (n=20 in total per group) according to the cement type: novel calcium silicate cement (Bond Protooth, Dentosolve, Aarhus, Denmark), zinc phosphate cement (DeTrey, Dentsply, Germany), and glass ionomer cement (Fuji I, GC, Tokyo, Japan). A small metal piece was soldered to the head of the titanium post (ParaPost XP, Coltene/Whaledent, USA) in order to be used as anchorage for the tensile pull-out force during the test.

**Figure 1.**
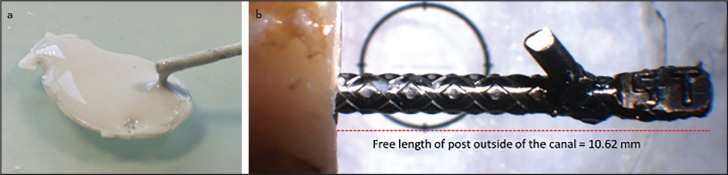
(a) Bond Protooth after mixing with creamy consistency, which is applied to the titanium post. (b) The measurement of the free length of metal post after cementation under stereomicroscope at ×10 magnification

A volume of 195-μl hydration liquid was pipetted to one gram pre-dosed Bond Protooth inside the mixing capsule and cap-mixed for 20 seconds (CapMixTM, 3M ESPE, Seefeld, Germany) to obtain creamy consistency (Fig. 1a). The other two cements were mixed according to the manufacturer’s instruction. After mixing, the paste was dispensed using a suitable spatula onto a plate. Each cement was placed into the canals using a #35 spiral lentulo attached to a slow-speed handpiece. Prefabricated titanium posts #5 (diameter=1.25 mm) (ParaPost XP, Coltene/Whaledent, USA) were coated with cement (Fig. 1a) and inserted into the canal. Excess cement was removed. In order to precisely measure the cemented length of the post, each specimen was photographed under stereomicroscope (Leica-Wild M420, Wetzlar, Germany) at ×10 magnification. The free length of the metal post outside of the canal was measured and subtracted from the total length of the metal post (19.4 mm) to obtain the cemented length of the post (Fig. 1b). The cemented area for each specimen was calculated based on cemented length of the post following the equation:

Cemented area (mm^2^)=side area+apical bottom of post=2π ×0.625 (radius of post)×cemented length of post+π (0.625)^2^= 3.927×cemented length of post+1.22718.

All specimens were stored in >95% humidity for 24 hours that was followed by immersion in phosphate-buffered saline (PBS) for 4 weeks at 37°C to allow strength development of the calcium silicate cement (19). A stainless-steel wire looped around the soldered metal piece head of the posts and then attached to the universal testing machine (Instron, Canton, MA, USA). The posts were subjected to increasing axial tensile force at crosshead speed of 2 mm/min. Before testing, the axial direction of posts was controlled to assure the tensile force loading along the post axis. The maximum load of bonding failure was recorded in Newton and divided into the cemented area of the post to calculate the pull-out bond strength in Mega Pascal (MPa).

### Statistical analysis

Shapiro-Wilk Test at significance level of 5% was used to check the normal distribution of the data. Data were analyzed by one-way ANOVA followed by Tukey post-hoc multiple comparisons at the significance level of 0.05 using statistical software (SPSS 21.0, IBM).

## Results

**Figure 2.**
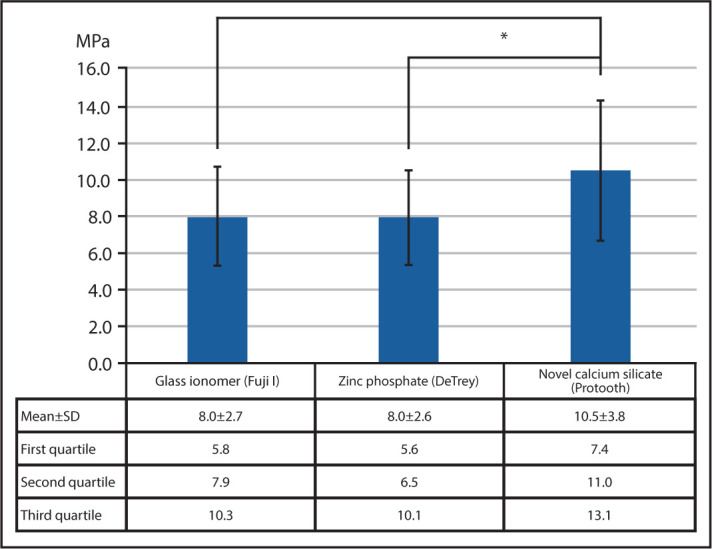
The means, standard deviations (SD), and interquartile ranges of pull-out bond strength of titanium posts cemented with novel fast-setting calcium silicate, zinc phosphate, and glass ionomer cements. *represents a statistically significant difference (P<0.05)

Figure 2 illustrates the means, standard deviations, and interquartile ranges of pull-out bond strength value of titanium posts cemented with novel calcium silicate cement (Bond Protooth), zinc phosphate cement (DeTrey), and glass ionomer cement (Fuji I). Three samples in the zinc phosphate group and one sample in the novel calcium silicate group experienced a detachment of the soldered head during testing and were discarded from the study. Shapiro-Wilk Test disclosed normal distribution of the data (P>0.05). One-way ANOVA revealed a statistically significant difference in pull-out bond strength between the groups (P=0.022). Novel calcium silicate cement showed a significantly higher pull-out bond strength compared to zinc phosphate (P=0.044) and glass ionomer (P=0.043) cements (Fig. 2).

## Discussion

The findings of this study revealed that retentive bond strength of intracanal posts cemented with novel fast-setting calcium silicate cement (Bond Protooth) was significantly higher than traditional zinc phosphate and glass ionomer cements after 4 weeks of immersion in PBS. So, the null hypothesis was rejected.

In this study, we used serrated parallel-sided titanium metal posts, which provide reliable retention (26), and lower risk of root fracture than tapered metal posts (27). Glass fiber-reinforced composite resin-based posts are used with resin-based luting cements (28), and they were not included in this study. Zinc phosphate and glass ionomer were selected as the comparison cement because zinc phosphate is always considered as standard for metal post cementation (18), and glass ionomer exhibited an acceptable clinical performance (27). Further studies to evaluate the bond strength of different post systems such as cast post-core or stainless-steel metal posts seem relevant.

In this study, we reported the pull-out strength in MPa by dividing the maximum load value to the cemented area of the post by considering the post geometry as an even cylindrical shape. This means that internal micro-area of the serrated grooves over the post structure was not included in the calculation. Since this geometry was completely similar in all specimens in the three experimental groups, this variation might have a limited influence on the findings of the study. Although the post space preparation and depth of post cementation standardized, small variations were inevitable. Accordingly, precise measurement of the cemented depth of the post was performed after cementation. The post cementation depth was measured under stereomicroscope for each sample (Fig. 1b) to provide data for accurate calculation of cemented area. The smear layer was removed before cementation in this study to improve the cement bonding ability to root dentine (29, 30). Although the Bond Protooth tested in this study does not contain additional zirconium oxide as radiocontrast material, its radio opacity is higher than dentine (unpublished data). Root canals with round cross-sections were only included in this study because the volume and thickness of cement would substantially vary depending on post geometry in oval shape canals (31).

Novel calcium silicate powder is mixed with 2% polycarboxylic weak acid as water-based hydration liquid. By precise adjustment of the liquid-to-powder ratio, the consistency of the new cement is controllable to obtain a suitable creamy consistency for cementation purposes (Fig. 1a). The liquid-to-powder ratio plays a critical role in the determination of the water pore volume degree, porosity, solubility, and eventually the mechanical properties of calcium silicate cements (32). Vargas et al. (18) reported that ProRoot MTA has an inferior retentive strength compared to glass ionomer and zinc phosphate cement when it was used as cementing agent for metal posts. They also reported a non-significant difference between glass ionomer and zinc phosphate cements, which is in agreement with findings of the current study.

Studies demonstrated that innovated calcium silicate cement (Protooth) has improved diametral tensile strength in comparison to ProRoot MTA (19), and higher bonding ability to dentine compared to ProRoot MTA and glass ionomer cement (21). This supports the finding of the current study proposing an improvement on properties of the earlier generation of calcium silicate cement (ProRoot MTA) that would potentially match and exceed the bond strength of traditional zinc phosphate and glass ionomer cements. 

The apatite-forming ability of calcium silicate cement contributes to biomineralization (20), dentine bonding enhancement (21), gap closure at the dentine-cement interface (20), and bacterial microleakage reduction (33). The presence of fluoride composition accelerates the apatite-forming ability of the cement (12). Induced high alkaline pH due to substantial calcium hydroxide release by calcium silicate cements during setting advocates the antibacterial properties to these cement (34), which is in contrast to acidic glass ionomer and zinc phosphate cements. Indeed, glass ionomer cements are able to chemically bond to tooth substrates (35) that tends to decrease overtime (21). Zinc phosphate cement does not provide a chemical adhesion and the retention basically relies on mechanical retention to rough surface irregularities (36). Interestingly, calcium silicate cements dentine-bonding mechanism has been attributed to formation of a bioactive interfacial calcium phosphate (i.e. apatite) layer, where calcium phosphate depositions penetrate into the dentinal tubules (37, 38), and improve the bond strength to dentine (14) and the cement sealing ability (33).

Eventually, all discussed properties of the novel cement favour its application as cementing agent that not only supports improved sealing in the root canal system, but also in perspective may support fluoride assisted caries control in the crown. Placement of intracanal posts in short canals or canals with perforation is challenging due to compromised sealing (39) or insufficient remaining space (40). The root repair material for perforation repair fills a substantial space in the root canal, which is normally available for post accommodation. So, placement of a shorter intracanal post is indicated that may lead to a reduction in retention or compromised sealing. In this scenario, a biocompatible material that would concurrently work as both repair material, by providing a good sealing and hard tissue regenerative capability, and also as a superior luting agent is clinically preferable (18). However, further studies investigating the physico-chemical properties and dimensional stability of the novel cement are needed.

The observation in this study on post pull-out bond strength, supported by earlier observations on sealing properties (20), biocompatibility (22-24), bonding to dentine (21), and apatite-forming ability (12), suggest that this novel calcium silicate is a potential alternative as a luting cement.

## Conclusion

Within the limitations of this study, the pull-out bond strength of titanium post cemented with Bond Protooth in root canal treated teeth was superior to zinc phosphate and glass-ionomer cements after 4 weeks immersion in PBS.

### Disclosures

**Conflict of interest:** The co-author Henrik Løvschall has a financial relationship with Dentosolve as patentee. The other authors declare that they have no financial relationship or conflicts of interest.

**Ethics Committee Approval:** According to the Danish National Committee on Health Research Ethics, this study did not require an ethical approval; anonymous biological materials (i.e. teeth that had already been removed for dental treatment reasons). https://en.nvk.dk/rules-and-guidelines/act-on-research-ethics-review-of-health-research-projects.

**Peer-review:** Externally peer-reviewed.

**Financial Disclosure:** This study did not receive any financial support.

**Authorship contributions:** Concept – H.L., B.R.; Design – H.L., B.R., M.L.; Supervision – B.R., H.L.; Funding - H.L.; Materials - H.L.; Data collection &/or processing – B.R., M.L.; Analysis and/or interpretation – B.R., H.L., M.L., A.V.; Literature search – B.R., A.V.; Writing – B.R., A.V.; Critical Review – B.R., H.L., M.L., A.V.
